# Asymmetrical slip propensity: required coefficient of friction

**DOI:** 10.1186/1743-0003-10-84

**Published:** 2013-07-31

**Authors:** Jung-suk Seo, Sukwon Kim

**Affiliations:** 1 Division of Sports Science, College of Natural Science, WonKwang University, Iksan-si, Jeollabuk-do, South Korea; 2 Department of Physical Education, College of Education, Chonbuk National University, Jeonju-si, Jeollabuk-do, South Korea

**Keywords:** Gait asymmetry, RCOF, Slip propensity, Limb asymmetry, Gait kinetics

## Abstract

**Background:**

Most studies in performing slips and falls research reported their results after the ipsilateral leg of subjects (either right foot or left foot) was guided to contact the contaminated floor surface although many studies indicated concerns for asymmetries of legs in kinematic or kinetic variables. Thus, the present study evaluated if dominant leg’s slip tendency would be different from non-dominant leg’s slip tendency by comparing the Required Coefficient of Friction (RCOF) of the two lower limbs.

**Findings:**

Forty seven health adults participated in the present study. RCOF was measured when left or right foot of subjects contacted the force platforms respectively. Paired t-test was performed to test if RCOF and heel velocity (HCV) of dominant legs was different from that of non-dominant legs. It was suggested that the asymmetry in RCOFs and HCV between the two lower limbs existed. The RCOFs of non-dominant legs were higher than that of dominant legs.

**Conclusions:**

The results indicated that asymmetry in slip propensity, RCOF, was existed in lower extremity. The results from the study suggested that it would be benefit to include a variable, such as asymmetry, in slips and falls research.

## Findings

### Introduction

Many studies have attempted to find mechanisms in association with slip-induced fall accidents. In most of these studies, 1) experimental protocols that mimicked slips were included and 2) characteristics of either right or left lower limbs while it was striking the floor materials were reported. Particularly, studies introduced postural perturbations while the subjects’ right foot was landing on the surface [1-8], *whereas*, another studies introduced perturbations while the subjects’ left foot was landing [9-11]. In addition, Gronqvist et al. [12] provided perturbations to either left or right lower limb when it was striking the floor.

RCOF (Required Coefficient of Friction) represents the minimum coefficient of friction that must be available at the shoe-floor interface to present slip initiation. RCOF was determined by the ratio of vertical ground reaction force over horizontal ground reaction force [13] during heel contact phase of gait cycle. *Assuming that the RCOF from the two lower limbs were different, one could expect that measuring the RCOF of the right lower limb in one study would produce different results than studies that measure the RCOF of the left lower limb of the same subjects.*

Dangerous slips are most likely to occur when RCOF at the shoe-floor interface exceeds the available coefficient of friction (ACOF) of the floor. Slip severity would increase as the difference between the RCOF and available dynamic COF of the floor surface increases [14,15]. It was suggested that lower limbs of healthy people with no injury behave asymmetrically while walking [16]. Studies suggested asymmetrical spatial-temporal and kinematic behaviors between dominant legs and non-dominant legs [17-20]. *The present study hypothesized that slip tendencies of the two lower limbs would be different.*

## Methods

**Figure 1:**
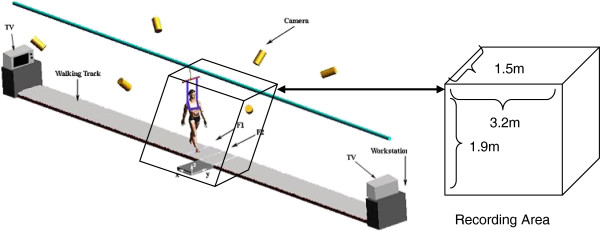
**Laboratory set-up (adapted from**[21]**).**

Forty seven healthy adults (20 females and 27 males, ages between 21–70 years old, 169.6±7.8 cm, BMI of 25.9±4.3) participated in this study. Some volunteers were excluded if they reported any physical problems affecting their walking. Each participant completed an inform consent procedure approved by the university’s Internal Review Board (IRB). Walking trials were conducted on a walking track (20 m), which elevated 15 cm above the floor surface (Figure 1, adapted from [21]). Two force plates (BERTEC # K80102, TYPE 45550–08) covered by the vinyl tile were used to collect ground reaction forces at 1200 Hz. The recoding area was about.

Each participant was asked to walk at his or her preferred walking speed. They were asked to walk freely and their start points at each end were adjusted until their walking was consistent throughout their test time. This allowed his or her left foot landed on the first force plate and the right foot landed on the second force plate in a row. For every participant, one RCOF of left heel contact was collected from the first force platform (F1 in Figure 1) and one RCOF of right heel contact was collected from the second force platform (F2 in Figure 1). Their kicking legs (soccer ball) were considered for dominant legs. There were only 2 participants who indicated their left leg for kicking leg.

The difference in RCOF and HCV of dominant leg and non-dominant leg was evaluated using paired t-tests (SPSS 12.0). The results were considered as statistically significant when p≤0.01. Table 1 compares mean and standard deviation of the RCOF in dominant (initial as “D”) and non-dominant (initial as “N”) legs. Paired-t test indicated that RCOF (t=4.61, 46, P < 0.0001) was statistically higher in dominant leg, HCV (t=12.24, 46, P < 0.0001) was statistically faster in non-dominant leg, and TA COM (t=3.21, 46, P = 0.0001).

**Table 1:** Descriptive statistics of RCOF, HCV, and TA COM

	**RCOF**	**HCV(cm/s)**	**TA COM(cm/s2)**
D	0.176±0.032	217.9±57.34	137.12±531.36
N	0.193±0.034	107.42±44.77	157.92±513.73

## Discussion

Changes in either vertical forces or horizontal forces may alter RCOF. Horizontal heel velocity (i.e. HCV) and forward acceleration of the whole body center of mass (TA COM) have been thought to increase the RCOF due to their effects on the variation in the horizontal force component [1,15,22].

One of principal actions of lower extremity muscles is to accelerate and to decelerate angular motions of the legs when walking [23] and Rice and Seeley [24] suggested that the non-dominant lower limb contributed more to support impulse (upward acceleration of COM), while the dominant limb contributed more to propulsion impulse (forward acceleration of COM) when walking fast. In the present study, higher RCOFs were found when the non-dominant legs were contacting the floor surface. Faster forward acceleration of the center of mass by the dominant lower limb could have resulted higher RCOF. By Newton’s 1^st^ law, acceleration profiles affects ground reaction force production since a person’s mass is constant. Faster TA COM could contribute to a relatively larger increase in horizontal ground reaction forces than vertical ground reaction forces resulting in higher RCOF. It was suggested that, when heel contact velocity was minimized to zero, the body traveled forward from one point to the other point in order to maintain forward body momentum [1,15]. As the stance limb prepares to leave the ground, the contralateral limb contacts ground and accepts the body’s forward momentum. As a result, the imposed horizontal force relative to vertical force could increase more at shoe-floor interface as the whole body COM travelled forward rapidly [1] contributing to an increase in RCOF when non-dominant leg was contacting the floor surface and the body was transitioning over non-dominant legs (i.e. ratio of horizontal force to vertical force).

In previous studies [22], faster heel contact velocity was suggested to increase RCOF, thus, the likelihood of slip-induced falls. Therefore, heel contact velocity may be one of factors contributing to a risk of slip-induced falls among the elderly [22]; Mills and Barrett [25]. However, the majority of slips and slip-induced falls were likely to occur 50–80 ms after heel contacted the ground (i.e. RCOF occurs at about 70 ms after heel contact) [1,4]. Accordingly, the suggestions by Winter et al. [22], and Mills and Barrett [25] should not be significant since heel velocity right after heel contact should be minimized almost to zero [1]. Thus, it may be unconvincing to suggest that RCOF, which occurs 70–120 ms after heel contact, may directly be related to the heel contact velocity.

Several studies suggested that as differences between the friction demand characteristic (i.e., RCOF) and available dynamic COF increased, the number of slip events would increase [13,14]. Dangerous slips occurred when the friction force opposing the movement of the foot was less than the shear force of the foot shortly after the heel contacted the ground [1,4,14]. Given the constant contact time and mass associated with heel contact of the gait cycle [26], the impulse-momentum relationship indicates that horizontal shear force increases more as the differences of the whole body COM velocity became larger. If the push-off forces of dominant legs were larger propelling the whole body COM faster, the horizontal shear force produced at the shoe-floor interface became larger proportionally, resulting in a larger RCOF of non-dominant limb.

The statistical differences in RCOF of the two limbs seen in the present study suggested that the previous studies [2,5,7,9-12,27] could come to different conclusions just because of kinematics of the two limbs. In addition, asymmetrical strength of the two legs [28-30], especially greater asymmetry in older adults [29], could influence kinematics of the either striking leg or supporting leg (rear leg). The leg strength and/or power was correlated to fall risk; fallers generated less leg strength or power [29,31-33] because the ability to generate an adequate neural response, thus, the power in muscles would be a key factor in producing movement and its control [4,29]. By analyzing the kinematic data, it is easily seen that the slipping leg would extend at knee joint while the support leg (real leg) would flex at knee joint. Hamstring muscle of slipping leg and rectus femoris muscle of rear leg should produce adequate eccentric contraction at knee joints in order to stop hyperextension of slipping leg or hyperflexion of supporting leg (i.e. out of normal range during walking) at knee joints. Without the proper generation of muscle power, a person would fall. Particularly, older adults showed difficulty in controlling activities when a strong eccentric component was required [34]. The asymmetrical eccentric strength could influence the likelihood of slip-induced falls, especially, in older adults.

In conclusion, measuring or evaluating the kinematics and kinetics of just one leg would have some limitations in making statements about the likelihood of slips and falls. Some people with greater asymmetry in lower extremity would exhibit a significant difference in the likelihood of slips and falls between the two legs. This would result in a significantly different outcome when interpreting data. Finally, there would be a substantial advantage if studies in regard to the slips and falls should consider including the variable, such as asymmetry.

## Competing interests

The authors declare that they have no competing interests.

## Authors’ contribution

SK and JS have made contributions to interpretation of data. SK and JS have been involved in drafting and revising the manuscript, and analyzing data. SK has been involved in acquisition of data, and development of conception. All authors read and approved the final manuscript.
